# Validation of a Smartphone Application for Monitoring Circadian Appetite: A Randomized Crossover Trial in Free-Living and Controlled Settings

**DOI:** 10.3390/nu17030384

**Published:** 2025-01-22

**Authors:** Shani Tsameret, Oren Froy

**Affiliations:** Institute of Biochemistry, Food Science and Nutrition, Robert H. Smith Faculty of Agriculture, Food and Environment, The Hebrew University of Jerusalem, Rehovot 76100, Israel; shani.tsameret@mail.huji.ac.il

**Keywords:** appetite, chrononutrition, smartphone application, pen and paper, circadian, meal

## Abstract

Background/Objectives: Appetite is affected by the circadian clock and exhibits diurnal variations. Nevertheless, accurately measuring subjective appetite throughout the day in a free-living setting poses a challenge. This study presents the validation of a novel smartphone application designed to capture, process and analyze circadian appetite fluctuations in a free-living setting. Methods: Twenty-four healthy participants (ages 38.34 ± 3.2 years) completed this randomized crossover study. Participants completed subjective appetite questionnaires every 30 min in a free-living environment, starting from waking hours until bedtime, using visual analog scale (VAS) via the pen and paper (PP) method and the new smartphone application (App). In addition, on two experimental days, separated by a week of washout, participants were provided either a high-energy breakfast (850 kcal) or a low-energy breakfast (250 kcal). Participants completed the appetite questionnaires using both methods every 30 min for 4 h, followed by an ad libitum lunch. Results: The high-energy breakfast led to significantly increased fullness sensation and decreased hunger, desire to eat, prospective consumption, and appetite score compared with the low-energy breakfast. There was no significant difference between the methods (PP and App) in all measured parameters under both the free-living environment and the controlled environment. Additionally, Bland–Altman analysis revealed a high degree of agreement between the PP and App methods. Moreover, most participants rated the App as the preferred method regardless of age. Conclusions: Our findings show that the new smartphone application is a valid, reliable tool for measuring subjective appetite, suitable for use in chrononutritional studies conducted in a free-living environment and controlled settings.

## 1. Introduction

A pivotal aspect of effective nutritional intervention lies in the participant’s ability to adhere to dietary changes for extended durations [1]. Compliance may be particularly challenging in free-living environments, as it necessitates ongoing patient cooperation and frequent face-to-face follow-ups for data collection. Food intake monitoring can be complex due to the high variability in human eating patterns, social life and lifestyle habits [2]. A free-living environment nutritional study can be even more challenging to comply with, as constant temptations and social pressures surround participants. This highlights the need to investigate appetite control and cravings around the circadian cycle. Accumulating evidence emphasizes the importance of meal timing as it may influence weight loss effectiveness and enhance metabolic benefits in healthy, obese and type 2 diabetes patients [3,4,5]. For example, it was shown that a meal timing schedule of three meals with the most energy and carbohydrates consumed in the early phase of the day, compared to a six-meal diet, led to weight loss and a significant reduction in hemoglobin A1c, appetite and overall glycemia, with a decrease in daily insulin dose [6].

Subjective appetite offers a practical, non-invasive and cost-effective alternative for assessing circadian appetite control, as opposed to the demanding and costly monitoring of biochemical factors, such as ghrelin, leptin and other appetite-related circulating factors [7]. Its simplicity and efficiency make it a valuable complement or standalone method for comprehensively gaining insights into the broader spectrum of circadian appetite regulation [8,9]. A frequently used tool for measuring subjective appetite is the pen and paper (PP) visual analog scale (VAS) method [10,11]. The questionnaire consists of appetite-related questions, presented on a 100 mm horizontal scale, anchored at each end with extreme responses. The VAS is usually provided to participants as a printed questionnaire, and they are asked to manually mark a vertical line on the scale, matching their subjective sensation at a given moment. Their answer is then calculated by manually measuring the distance from the left anchor of the horizontal line of the scale to the marked response [11].

While the VAS is a commonly used tool, its manual data collection process poses limitations, especially in large-scale or circadian and metabolism studies, as many time points should be monitored daily, which may lead to decreased compliance rates and potential data loss. Moreover, in the post-COVID-19 era, a paradigm shift towards hybrid lifestyles, marked by increased reliance on virtual gatherings and meetings, has been observed among individuals and workplaces, including healthcare and research facilities [12]. This change has emphasized the need for new and creative ways to collect data for clinical research using virtual methods, while ensuring data security and privacy.

To provide solutions to the limitations of PP methods, several electronic alternatives [13,14,15] (Electronic Appetite Rating System (EARS)), including smartphone applications [16,17] have been developed. Over the years, alterations in the operating system, screen and scale size, graphics and methodology were made, resulting in newer devices, which were validated compared with the pen-and-paper method [13,14,18], or compared to the previous EARS [15,19]. A few smartphone applications were developed for the assessment of the VAS; all were validated compared to the PP method. Hernandez-Morante et al. [17] developed a smartphone application for the Spanish-speaking population, using both free-living and laboratory settings. Holliday et al. [16] validated their APPetite application by using a thoroughly planned test–retest study design in a free-living environment without applying any nutritional intervention. Their VAS comprised an 11-point Likert scale [16]. A recent smartphone application validated in a free-living setting included 4 h of subjective appetite recordings after consumption of a fixed pre-shipped 230 kcal or 460 kcal breakfast [20]. These analyses included a continuous VAS with a slider on top of the horizontal line [20]. The different smartphone applications were validated using different scales, languages, research settings and diets, which are not necessarily compatible with the use in chrononutrition studies.

Contrary to English or Latin language, the Israeli population uses right-to-left languages, namely Hebrew and Arabic, which can affect the script, layout and navigation of the application. Using an English language questionnaire in a non-native English-speaking population requires a broader understanding of the language both semantically and culturally. Therefore, we found it essential to develop a tailored research tool, easy to use via the participants’ personal smartphones, that would be validated for use in chrononutrition studies. We developed a smartphone application to analyze subjective circadian appetite. The smartphone application is designed to utilize the process of data gathering and calculation by performing an automatic analysis once inputs are submitted by the user (with the addition of automatic time and date stamps) while maintaining privacy and data security. It is an eco-friendly, easy-to-use alternative while minimizing research bias and saving time and costs. Another goal was to ensure our smartphone application was adjusted to diverse age groups through intuitive design and ease of use. This is extremely important due to the continuous rise in lifespan and in the proportion of older adults with chronic illnesses [21]. As older adults are a vital study population in clinical research, we expected that creating a user-friendly experience might enhance compliance, specifically in chrononutrition free-living environment studies. To validate the smartphone application (App) in comparison to the gold standard pen-and-paper (PP) method, we conducted a crossover study under controlled laboratory and free-living environments in healthy participants.

## 2. Materials and Methods

### 2.1. Translation

The questionnaire was validated in Hebrew using the back-translation method. The questionnaire consisted of five questions regarding subjective appetite sensations. It was first forward-translated from English to Hebrew by two independent translators from the research group. The translations were then reconciled to a single forward translation in Hebrew, which was then back-translated into English by two independent translators, fluent in Hebrew and English and unfamiliar with the questionnaire. The next step included harmonization of the questionnaire by the primary investigator and the leading researcher. The latter version was then introduced to a panel of experts in biochemistry, food, and nutrition. The revised questionnaire was then used for cognitive debriefing interviews with ten volunteers fluent in Hebrew and English.

### 2.2. Participants

Twenty-four healthy volunteers (fifteen men and nine women) were recruited to this study by means of advertisements. The study population intentionally included a wide age range, including young and older adults, to assess the subjective reliability and comfort of use of the new method in the different age groups. Participants in the controlled setting were at a mean age of 38.3 years old, with a mean body weight of 75.6 kg and a mean BMI of 25 kg/m^2^ (Table 1). Participants in the free-living setting were at a mean age of 30.3 years old, with a mean body weight of 76.7 kg and a mean BMI of 25.7 kg/m^2^. Exclusion criteria included those who were pregnant or lactating, had any clinical illness such as diabetes, kidney or liver disease, or were taking any medications affecting body weight, food intake, or appetite. Vegan, vegetarian or celiac participants or any participants suffering from food sensitivity or food allergy were also excluded. Each participant was interviewed by a clinical dietitian to assess compatibility with this study and gave their written informed consent. The Institutional Review Board (IRB) of the Hebrew University of Jerusalem granted ethical permission for this study.

Results are mean ± standard deviation and range (minimum value–maximum value). NS (non-significant). Group 1 and Group 2 refer to the division of participants according to the crossover design. Group 1 participants started with the high-energy breakfast meal (HEB) test and after a week of washout proceeded to the low-energy breakfast (LEB). Group 2 participants started this study with the low-energy breakfast (LEB) and after a week of washout proceeded to the high-energy breakfast (HEB).

### 2.3. Study Design

**Figure 1:**
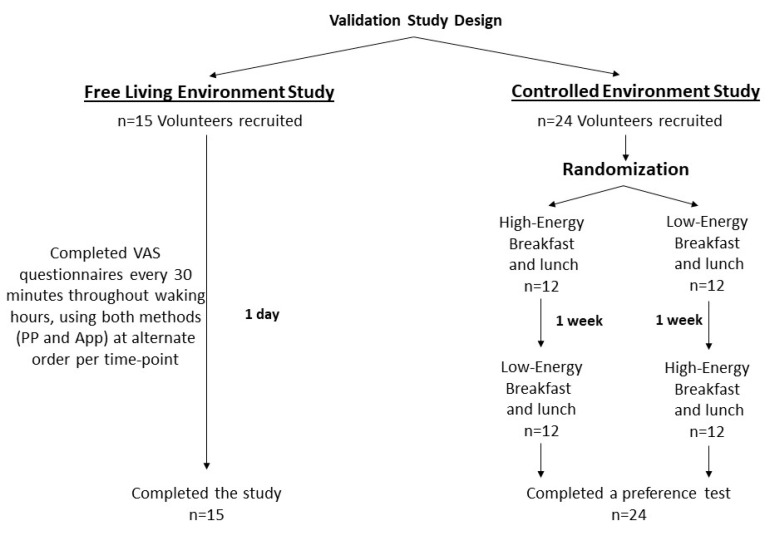
Study design.

This study comprises a free-living setting experiment and a controlled setting experiment (Figure 1). For the free-living environment experiment, participants were asked to assess their appetite sensations every 30 min during waking hours using both methods, in alternate order for each time point throughout the day. For example, upon awakening, participants were instructed to complete the questionnaire via the smartphone application and then proceed to the PP method. Noteworthily, after submitting each question, the smartphone application would block the option to go back and enable the user only to proceed to the next question. Participants were also instructed to change the order of the methods in use between time points. For example, if at 8 AM a subject started with the smartphone application, the following time point would start with the PP questionnaire. The participants were a subset of the controlled environment experiment (see below).

The controlled setting experiment included a within-subject crossover study, using time (11 time points), meal (high-energy breakfast or low-energy breakfast) and method (pen and paper vs. smartphone application) as independent factors. All twenty-four subjects were evaluated in two experimental days, separated by a minimum of 7 days. Each participant was randomly assigned to a treatment order using a coin flip, determining whether they first received the high-energy breakfast (HEB, *n* = 12) or the low-energy breakfast (LEB, *n* = 12). All participants subsequently underwent identical experimental procedures for both meal tests (Figure 1). All participants were first interviewed and weighed by a registered dietitian. Subjects were asked to arrive at the meal test experiment at 8:00 AM, after an overnight fast of 8 h (water was permitted), and appetite scores were completed at baseline (time −30). On the day of the experiment, participants stayed seated in their place. They were allowed to work on their laptops, read and speak to one another as long as no food-related subjects were discussed or observed. Both breakfasts (HEB or LEB) were served, and participants completed the questionnaires every 30 min for four hours. Lunch was then served after weighing the plates to adjust the portion size as described in Table 2. Participants were instructed to eat until feeling subjectively full and were encouraged to take additional food if desired.

Participants were instructed not to change anything in their ordinary routine, including physical activity or diet, the week prior to each experiment day and during the days of the free-living setting experiment. Previous studies have demonstrated that a sample size of *n* = 12 is sufficient to detect a within-subject difference > 10 mm, a criterion suitable for detecting variations in appetite ratings due to technical or physiological differences [11]. However, these studies included a narrow range of patient characteristics (for example, age and weight). As we intend to validate our smartphone application for use in the general healthy population, in both free-living and laboratory settings, as well as in two age groups, we chose a sample size of *n* = 24 participants. This study was conducted according to the Equator Network guidelines. Specifically, it was conducted and reported in accordance with the CONSORT 2010 guidelines and their extension for randomized crossover trials.

### 2.4. Diets

The fixed breakfast was similar to a breakfast given in a high-energy breakfast diet as described in previous work [6,22]. The high-energy breakfast included 850 kcal, approximately 40–55% of the daily caloric intake in a 1700–2000 kcal energy diet. The diet consisted of 42% carbohydrates, 28% protein and 30% fat, mainly from yogurt, cheese, toasted bread rolls, milk and oats. The low-energy breakfast (LEB) was ~3.5 less dense and included 250 kcals, which is ~15% of a 1700–2000 kcal daily energy intake (48% carbohydrates, 30% protein, 22% fat), mainly from bread and spreadable cheese. The vast caloric difference between the two diets was chosen to show the expected distinct differences in appetite scores and validate the new method for assessing appetite in different diets, as part of chrononutrition studies. Ad libitum lunch was served 4 h after the fixed breakfast. Lunch was prepared by the same caterer under the same conditions in the evening prior to each experiment day. An average meal contained a mean caloric intake of 750 kcals (40% carbohydrates, 24.3% protein, 33.8% fat) and consisted of mashed potatoes, roasted salmon, green beans, and fresh vegetables. Participants were instructed to eat until they felt comfortably full. The macronutrients composition of each breakfast and lunch provided to participants is shown in Table 2.

### 2.5. Subjective Appetite Scores

The subjective appetite scores were measured by a visual analog scale via pen and paper (PP) and a new smartphone application (App). Participants were instructed to download the application to their own smartphone devices. The VAS included five questions for the assessment of subjective appetite (Supplementary Table S1). Additionally, to assess the average daily general appetite, the individual daily mean score of each subjective appetite parameter in each method was analyzed using the following calculation: Appetite score = [desire to eat + hunger + (100 − fullness) + prospective consumption]/4. All questions were accompanied by horizontal lines anchored at each end by words describing the extreme sensations. The smartphone application presented the questions automatically in landscape mode, as each question was presented on a designated screen, which included the question, the scale and a dynamic cursor along the horizontal line. The cursor was moved by the finger without directly touching the horizontal line, thus removing an unwanted answer entered by accidentally touching the screen. Only after the cursor was moved to the desired point on the horizontal line, a “continue” button became accessible to confirm the response and proceed to the next question. Once the question was answered, subjects could not return to the previous screen. Participants completed the two methods in alternate order, attending to the Latin square. As they completed one method, the questionnaire was removed (i.e., exit the app or remove the printed questionnaire) to ensure no bias or comparison of their responses with the other method. VAS scores obtained from the PP method were measured twice by the research team to ensure the repeatability and reliability of each given value. VAS scores of the smartphone application method were automatically calculated and downloaded from the application’s secure server. Participants were asked to rate their experience regarding the preference, reliability, modernization and ease of use of each VAS method provided to participants at the end of each measurement day.

### 2.6. Statistical Analysis

The equivalence of the PP and APP methods was defined as an absolute difference of no more than 5 mm (5%). Bland–Altman plots were used to assess equivalence visually [23]. The Two One-Sided TTest (TOST) was used to calculate the *p*-value for equivalence. Individual preference questions were compared between age groups by the Fisher exact test. The area under the curve (AUC) was calculated with the trapezoid method [24]. Pearson correlation coefficients were calculated between the AUC of PP and App methods for the “hunger”, “fullness”, “desire to eat”, “prospective consumption” and “desire for sweets” variables. A one-way ANOVA (time of day) test was performed to analyze the circadian pattern of subjective appetite parameters with several time points. The Medcalc application was used to create Bland–Altman plots. SAS (version 9.4) was used for all other calculations. *p* values less than 0.05 were considered statistically significant.

## 3. Results

### 3.1. Validation of the Smartphone Application vs. Pen and Paper in a Free-Living Environment

**Figure 2:**
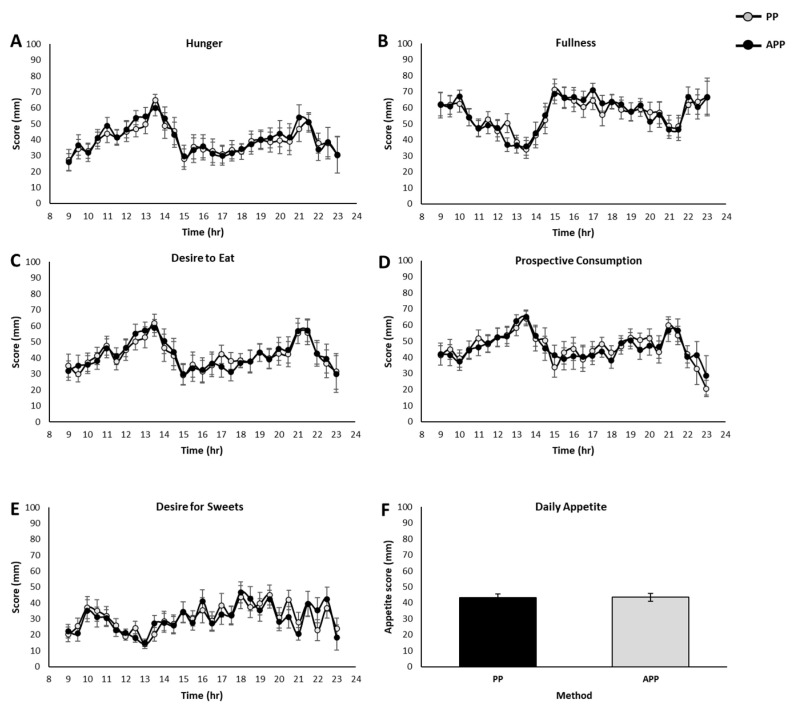
Circadian appetite in free-living environment settings. Graphs show the comparison of the pen-and-paper method and the smartphone application for appetite parameters: (**A**) Hunger. (**B**) Fullness. (**C**) Desire to eat. (**D**) Prospective consumption. (**E**) Desire for sweets. (**F**) Daily appetite score. Data presented as mean ± SE.

**Figure 3:**
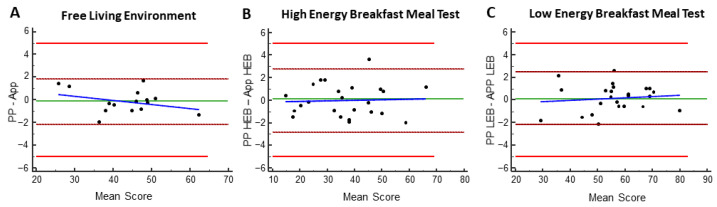
The Bland–Altman analysis. The Bland–Altman analysis showing the bias, limits of agreement and ± 1.96 x SD values of the appetite scores under free-living conditions (**A**) and of the high- or low-energy breakfast provided in the controlled setting meal test (**B**,**C**), measured by both paper-and-pen (PP) and smartphone application (App) methods. The Y-Axis represents the difference between the scores of the different methods (PP minus App). The X-Axis represents the mean appetite score of the App and PP methods. The red lines represent the limits which were defined as a 5 mm difference between the methods. The dashed burgundy lines represent the 95% confidence interval of ±1.96 SD; the green line represents the mean score. The dashed blue line represents the correlation. Each dot represents the difference between the methods for each participant.

There was no significant difference between the methods (PP and App) in hunger, fullness, desire to eat, prospective consumption and desire for sweets (*p* > 0.05, Student’s *t*-test) (Figure 2A–E). The daily average appetite score was also identical, as no changes were observed between the methods (*p* > 0.05) (Figure 2F). The Bland–Altman analysis revealed that all points fell within the 95% confidence interval for all parameters, reflecting a high degree of agreement between the PP and App methods (Figure 3A). These results indicate that the new smartphone application is as sensitive and reliable for the analysis of appetite and hunger as the gold standard method of pen and paper and can be used in studies involving free-living conditions.

### 3.2. Preference for a Smartphone Application over Pen and Paper

We next determined whether age affects preference by analyzing the results according to age [young adults (ages 24–31 years) and older adults (ages 53–65 years)]. Of the young adults, only one participant chose the PP as their preferred method (6.25%) (Table 3). All older adult participants but one chose the smartphone application as their preferred method (87.5%), and one did not have a preferred method (12.5%). There was no difference between the two age groups regarding the preference (*p* = 0.283, Fischer test), reliability of the method (*p* = 0.687, Fisher test), modern perception (*p* = 1, Fisher test) or ease of use (*p* = 0.423, Fisher test).

### 3.3. Oscillation of Appetite Sensations in a Free-Living Environment

The different appetite sensations presented rhythmic patterns (*p* < 0.05 ANOVA) (Figure 2A–E). Hunger, desire to eat and prospective consumption presented two peaks towards lunch and dinner, whereas desire for sweets, although maintaining relatively low scores throughout the day, gradually increased and reached its peak at ~18–20 h, compared with the lowest point around noon, similarly to previous publications [6,25]. These results indicate that the new smartphone application can monitor circadian appetite and hunger reliably in a free-living setting.

### 3.4. Effect of a Meal Test on Circadian Appetite Sensations

**Figure 4:**
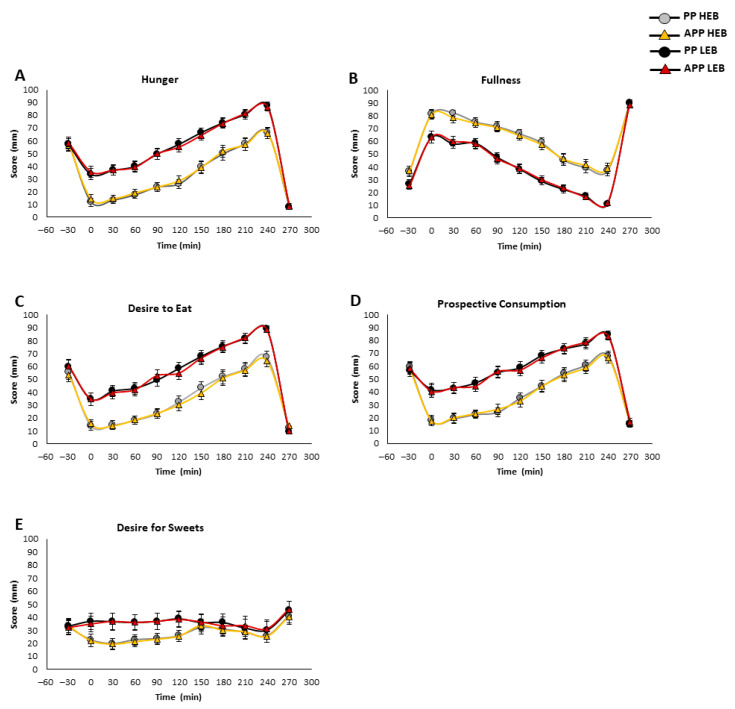
Subjective appetite after high- or low-energy breakfast. The effect of high-energy breakfast (HEB) and low-energy breakfast (LEB) on subjective appetite parameters. Graphs show results obtained by the smartphone application (App) and by the pen-and-paper (PP) method. The Y-axis represents the mean score (mm), the X-axis represents the time, (−30) refers to the baseline score before breakfast consumption. Time (0) represents scores obtained instantly post-breakfast. The different appetite parameters include the following: (**A**) Hunger. (**B**) Fullness. (**C**) Desire to eat. (**D**) Prospective consumption. (**E**) Desire for sweets. Data presented as mean ± SE.

We next conducted a cross-over meal test experiment with either high-energy breakfast (HEB) or low-energy breakfast (LEB), followed by lunch after 270 min, as was previously described [6,22,26]. The mean score (mm) was calculated per meal type (HEB or LEB) regardless of the order in which it was consumed by the participants. Figure 4 shows the results of both meal tests received by the smartphone application and pen-and-paper method. After breakfast, hunger, desire to eat and prospective consumption sensations gradually kept rising until reaching a peak after 240 min and a nadir after lunch (270 min) (Figure 4A,C,D). These sensations showed significantly reduced scores after HEB compared to LEB. Fullness scores increased immediately after breakfast, gradually decreased until reaching a nadir prior to lunch, and then peaked again after lunch (Figure 4B). Fullness showed a significant high score after HEB compared to LEB. Desire for sweets was significantly reduced an hour and a half after the HEB compared to the LEB (Figure 4E). Similar results were achieved using pen and paper (*p* > 0.05, Student’s *t*-test) (Figure 4A–E). Correlation coefficients were calculated for the area under the curve (AUC) for the appetite variables under the two different meal tests. Both methods showed very high correlations and were similar between the two breakfasts provided (Pearson correlation coefficients all *p* < 0.0001 and R^2^ > 0.97, Supplementary Table S2). Similarly, to the results received in the free-living settings, the Bland–Altman analysis revealed a high degree of agreement between the PP and App methods for the HEB and LEB meal tests (Figure 3B,C).

## 4. Discussion

In this study, we show that a smartphone application is a reliable tool for VAS measurement and easier to use compared with the pen-and-paper methodology. This study shows no difference between the methods in all appetite parameters under both free-living environments and controlled settings. In addition, a preference test showed that most participants, regardless of their age, rated the smartphone application as their preferred method and found it more reliable, modern and easy to use.

The area under the curve (AUC) of fullness scores showed a significant inverse correlation to the AUC of hunger sensation in the free-living environment experiment and after both LEB and HEB meal tests (Supplementary Figure S1A,B). As expected, after the ingestion of a very high-energy, high-protein and high-carbohydrate breakfast, all appetite measurements were significantly lower compared to the low-energy breakfast measurements, specifically the AUC of hunger, desire to eat and appetite score compared with the LEB. These results are supported by recent findings showing that consumption of a more extensive meal early in the morning significantly lowers average daily hunger or desire to eat [27]. The Bland–Altman analysis results indicate that the new smartphone application is as sensitive and reliable for analyzing appetite and hunger as the gold standard pen-and-paper method and can be used in studies involving free-living conditions and controlled settings.

We found that participants reported higher sweets cravings toward the evening under the free-living conditions, similar to recent publications [6,25]. The results reflect the general sensations regardless of food intake, as participants were not under any dietary restrictions or guidance and were able to fulfill their cravings by snacking or eating at any time, which could explain the relatively low desire for sweets observed in the free-living setting. Studies in the field of chrononutrition suggest that the circadian timing of energy consumption, as well as macronutrient distribution throughout the day, can affect energy utilization, glucose metabolism, weight loss, aging and disease risk factors [6,28]. The increasing evidence of the importance of circadian appetite and meal timing supports the notion that new smartphone applications need to be validated under feeding regimens used in chrononutrition studies, such as the high-energy breakfast provided in the meal test. Contrary to the measurement of general appetite in controlled settings, circadian appetite measurement can be more challenging. It requires higher real-time engagement, by recording many time points throughout the circadian cycle.

Recently, several new smartphone applications for the measurement of subjective appetite were developed and validated in free-living settings. Holliday et al. [16] validated the APPetite application in twenty-two participants in a free-living, cross-over design, in which participants recorded their subjective appetite every hour for twelve hours upon waking. However, their app consisted of an eleven-point Likert scale. As opposed to the Likert scale, a continuous 100 mm VAS scale may provide higher sensitivity to the results, since an undefined broader range is possible. Zhu et al. [20] validated a smartphone application in 102 participants; their free-living settings included four hours of subjective appetite recordings after consumption of a fixed pre-shipped 230 kcal or 460 kcal breakfast. The free-living settings in this study imitated the real-life environment of subjects participating in nutritional circadian studies, as they were asked to monitor their subjective appetite in a continuous analog scale in their natural environment, throughout the day, using the application in their smartphone device and by pen and paper.

During the past two decades, it seemed as though the Electronic Appetite Rating System (EARS) became the new gold standard compared to the pen-and-paper method, as researchers validated new electronic hand-held devices with the EARS [15,19]. However, recent publications of appetite VAS applications have been validated in free-living conditions compared to the pen-and-paper method [8,16,20,29]. This could stem from the extensive use of smartphones, making the use of alternative electronic devices almost irrelevant or impractical due to higher research costs and logistics of shipping. Overall, it seems that nowadays, using EARS devices does not differ much compared to the limitations of working with the pen-and-paper method, which provides a cheaper, immediate, and more accessible alternative.

One limitation of this study is the absence of a test–retest component to assess potential variations between the smartphone application and the pen-and-paper method for VAS measurement across multiple time points. Nevertheless, the reliability was validated by the Bland–Altman analysis alongside consistent and strong similarities during the two meal tests and free-living settings. Another limitation is the wide range of BMI. However, several studies show that neither BMI nor weight concerns were significantly associated with appetite ratings as measured by pen-and-paper VAS [30,31]. Moreover, Aukan et al. showed that plasma concentrations of gastrointestinal tract hormones, representing objective metabolic appetite sensors, did not differ among obesity classes except for insulin [31]. In light of these findings and the fact that none of the participants showed altered glucose metabolism, we did not find the BMI range critical. Additionally, our study design included the assessment of subjective appetite sensations using both PP and the App on the same day. Although a comparison of the results using the PP or the App on different days might have been more reliable, we would have had the problem of day-to-day variability in appetite, alongside many other uncontrolled parameters. In addition, our power calculations reassured us that our chosen sample sizes were sufficient to validate the smartphone application against the pen-and-paper method in both free-living and controlled environments. Nonetheless, we recommend that future studies consider larger and more diverse populations to further confirm the reliability and broader applicability of these findings. Future research could also explore the use of the Mobile App Rating Scale (MARS) for a systematic evaluation of the App’s engagement, functionality, aesthetics and informational quality [32].

Furthermore, our study focused solely on the meal test until the provided ad libitum lunch. Expanding the meal test to include dinner within controlled conditions could provide a more comprehensive understanding of the implications of HEB vs. LEB for circadian appetite. Exploring the desire for sweets during the evening, a period known for heightened snacking tendencies and increased cravings, could offer valuable insights.

## 5. Conclusions

Our findings show that our smartphone application is a valid, reliable tool for measuring subjective appetite by the visual analog scale, suitable for use in chrononutritional studies conducted under free-living environments and controlled settings. Understanding appetite regulation in real-world settings provides insights into the factors that influence eating behavior, such as social cues, stress and accessibility of food. These insights are crucial for developing personalized and context-specific strategies to promote healthy eating habits. They also highlight the role of environmental factors, such as food marketing, availability of nutrient-dense versus energy-dense foods and cultural norms around portion sizes, in shaping appetite control. Monitoring circadian appetite can help researchers and healthcare professionals better understand the underlying drivers of eating behaviors, enabling them to address barriers to compliance with health interventions and support long-term adherence to healthier lifestyle choices.

## Figures and Tables

**Table 1:** Patient characteristics at baseline.

	Free-Living Environment Study (*n* = 15)	Controlled Environment Study (*n* = 24)	Group 1 (*n* = 4 Female)	Group 2 (*n* = 5 Female)	*p* Value
Age (years)	30.3 ± 9.4 (24–63)	38.34 ± 15.72 (24–65)	37.25 ± 16.05 (25–64)	40.42 ± 15.94 (24–65)	NS
Weight (kg)	78.67 ± 17.11 (48–113)	75.65 ± 15.64 (48–113)	74.71 ± 15.22 (48–95)	76.58 ± 16.66 (56–113)	NS
Height (m)	1.74 ± 0.12 (1.58–1.93)	1.73 ± 0.11 (1.58–1.93)	1.72 ± 0.11 1.58–1.93)	1.75 ± 0.12 (1.58–1.9)	NS
BMI(kg/m^2^)	25.67 ± 3.9 (19.23–33.51)	25.05 ± 3.37 (19.23–33.51)	25.12 ± 2.66 (19.23–29.39)	24.99 ± 4.09 (20.82–33.51)	NS

**Table 2:** Nutrient composition of diet.

	Low-Energy Breakfast (LEB)	High-Energy Breakfast (HEB)	Lunch
Energy (kcal)	250	850	750
Carbohydrates (g)	30	90	75
Protein (g)	19	60	46
Fat (g)	6	28	28

**Table 3:** Preference test according to age groups.

Question	Age		
	Young Adults *n* = 16	Older Adults *n* = 8	All *n* = 24
Preferred method (%)			
Pen and paper (PP)	6.25	0	4.17
Smartphone application (App)	93.75	87.50	91.67
No preference	0	12.50	4.17
Reliability of the method (%)			
Pen and paper (PP)	6.25	0	4.17
Smartphone application (App)	62.50	87.50	70.83
No preference	31.25	12.50	25.00
Modernization of the method (%)			
Pen and paper (PP)	0	0	0
Smartphone application (app)	93.75	100.00	95.83
No preference	6.25	0	4.17
Ease of use			
Pen and paper (PP)	6.25	12.50	8.33
Smartphone application (App)	87.50	87.50	87.50
No preference	6.25	0	4.17
Percentage of Questions with Answer of “App”			
Mean	84.38	90.63	86.46
Std	25.62	18.60	23.29
Min	0.00	50.00	0.00
Q1	75.00	87.50	75.00
Median	100.00	100.00	100.00
Q3	100.00	100.00	100.00
Max	100.00	100.00	100.00

## Data Availability

Data will be made available on request. The data are not publicly available due to privacy and ethical reasons.

## Supplementary Materials

The following supporting information can be downloaded at https://www.mdpi.com/article/10.3390/nu17030384/s1: Figure S1: Hunger and fullness VAS scores in controlled settings; Table S1: The VAS questions. Table S2: Correlation Coefficients between pen and paper (PP) and the smartphone application (App) for all appetite parameters.
