# Supplementary material for: Validation of a Smartphone Application for Monitoring Circadian Appetite: A Randomized Crossover Trial in Free-Living and Controlled Settings
Source: Nutrients. 2025 Jan 22;17(3):384. doi: 10.3390/nu17030384 (PMC11820149; doi:10.3390/nu17030384)
Supplement: Supplementary file 1 [file nutrients-17-00384-s001.zip › nutrients-3430024-SI.pdf]

Supplementary Table S1: The VAS questions

	Question
**Hunger**	How hungry do you feel?
**Fullness**	How full do you feel?
**Desire to eat**	How strong is your desire to eat?
**Prospective consumption**	How much do you think you could eat?
**Desire for sweets**	Would you like to eat something sweet?

Supplementary Table S2: Correlation Coefficients between pen and paper (PP) and the smartphone application (App) for all appetite parameters

	High Energy Breakfast		Low Energy Breakfast	
	R^2	P value	R^2	P value
**Hunger**	0.988	<.0001	0.978	<.0001
**Fullness**	0.986	<.0001	0.967	<.0001
**Desire to eat**	0.972	<.0001	0.9906	<.0001
**Prospective consumption**	0.994	<.0001	0.991	<.0001
**Desire for sweets**	0.995	<.0001	0.998	<.0001

Supplementary Figure S1: Hunger and Fullness VAS scores in controlled settings

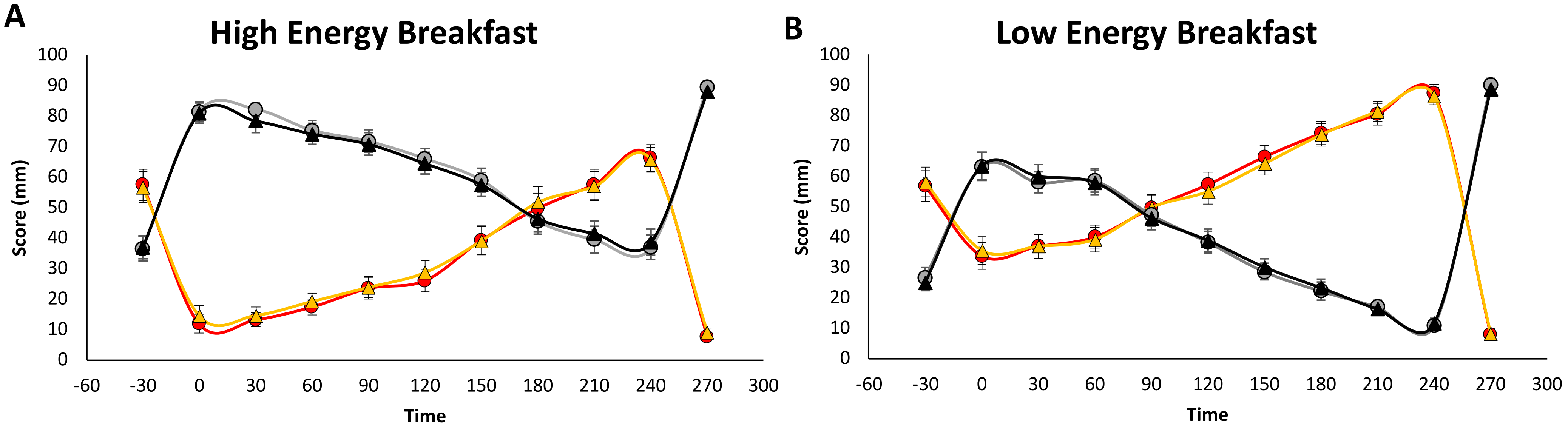

The area under the curve (AUC) of fullness and hunger parameters presented as measured by both methods. (A) Hunger and fullness parameters after ingestion of a high energy breakfast. (B) Hunger and fullness parameters after ingestion of low energy breakfast.
